# Productivity and sustainability of rainfed wheat-soybean system in the North China Plain: results from a long-term experiment and crop modelling

**DOI:** 10.1038/srep17514

**Published:** 2015-12-02

**Authors:** Wei Qin, Daozhong Wang, Xisheng Guo, Taiming Yang, Oene Oenema

**Affiliations:** 1 Soil and Fertilizer Research Institute, Anhui Academy of Agricultural Sciences, Hefei, 230031, China; 2 Key Laboratory of Nutrient Cycling and Resources Environment of Anhui Province, Hefei, China; 3 Anhui Center of Agricultural Meteorology, Hefei, 230031, China; 4 Department of Soil Quality, Wageningen UR, 6700 AA, Wageningen, the Netherlands; 5 Alterra, Wageningen UR, 6700 AA, Wageningen, the Netherlands

## Abstract

A quantitative understanding of yield response to water and nutrients is key to improving the productivity and sustainability of rainfed cropping systems. Here, we quantified the effects of rainfall, fertilization (NPK) and soil organic amendments (with straw and manure) on yields of a rainfed wheat-soybean system in the North China Plain (NCP), using 30-years’ field experimental data (1982–2012) and the simulation model-AquaCrop. On average, wheat and soybean yields were 5 and 2.5 times higher in the fertilized treatments than in the unfertilized control (CK), respectively. Yields of fertilized treatments increased and yields of CK decreased over time. NPK + manure increased yields more than NPK alone or NPK + straw. The additional effect of manure is likely due to increased availability of K and micronutrients. Wheat yields were limited by rainfall and can be increased through soil mulching (15%) or irrigation (35%). In conclusion, combined applications of fertilizer NPK and manure were more effective in sustaining high crop yields than recommended fertilizer NPK applications. Manure applications led to strong accumulation of NPK and relatively low NPK use efficiencies. Water deficiency in wheat increased over time due to the steady increase in yields, suggesting that the need for soil mulching increases.

Rainfed agriculture covers 80% of the world’s cultivated land and produces 60% of total crop production^1^. The relatively low productivity in rainfed agriculture is often due to limited water and nutrient availability, degraded soils, and poor water and nutrient management^2^,^3^,^4^. Forecasts suggest that food production will have to double in order to meet the demands of the expected 9–10 billion people in 2050^5^. A large fraction of this increase has to come from rainfed agricultural systems. Achieving this production target will increase the pressure on land, fresh water and nutrient resources unless these resources are used much more efficiently^6^,^7^,^8^. The pressure will likely become more severe under climate change, when more extreme weather events may occur, such as droughts^9^.

Water and nutrients are key factors for plant growth and development as they are involved in many processes in plants, including photosynthesis, respiration, transpiration, plant development, and yield formation^10^,^11^,^12^. There are also possible interactions between water and nutrient use in crop yield; water stress may lead to stomata closure, which inhibits nutrient uptake by the plant^13^,^14^,^15^. Plants with nitrogen (N) deficiency are often small and develop slowly, because of low efficiency in photosynthesis and plant development; as a consequence evaporative losses are relatively high and water use efficiency low. Adequate water and nutrient supply contribute to shoot and root growth, which increase plant water and nutrient uptake, and thereby yield^16^,^17^.

Most of the water and nutrients are taken up by the crop from the soil, and soil quality or soil fertility is a major determinant of the productivity of the land^18^. A combination of soil physical, chemical and biological characteristics defines how much water and nutrients can be stored in soil and how well these can be taken up by roots to meet the demands of the growing crop during the crop growing season. Farmers regularly apply fertilizers, manures, lime and crop residues to soils to improve the physical, chemical and biological quality and productivity of the soil. There has been a long standing debate whether chemical fertilizers alone can sustain soil quality and crop productivity over time^19^,^20^. Common view is now that chemical fertilizers can sustain crop productivity, provided all essential nutrients are supplied in adequate amounts and sufficient organic carbon is returned to the soil to replenish the decomposition losses. The remaining question is often how much nutrients have to be applied, and in which proportions, to sustain crop productivity and minimize environmental effects associated with nutrient losses.

This is a key question in the North China Plain (NCP), the most important food production area in China producing ~50% of the total national wheat and maize production^21^,^22^. Crop rotations are common practice in NCP, mostly winter wheat followed by summer maize or soybean^23^,^24^. Though animal density is high and animal manure abundantly available, and despite China’s long history of recycling of manures and wastes, almost no animal manure is being used in wheat and maize production during the last decades (but in vegetable and fruit production)^25^,^26^. Instead, wheat and maize are heavily fertilized with mineral NPK fertilizers, and because of its liberal use, losses are relatively high^27^. Questions were raised about the sustainability of these practices, which led to the initiation of long-term field experiments in NCP some 30 years ago. In these trials, combinations of chemical fertilizers with straw and animal manures were tested for underpinning fertilizer recommendations.

Water is also a limiting factor for crop production in the NCP, especially for double cropping systems that often require more water than mono cropping systems^26^,^28^. Currently, 70% of the winter wheat in the NCP is irrigated, mostly with groundwater, which has led to severe groundwater depletion (decline by 1 m per year)^29^,^30^. It is still unclear whether rainfed double cropping systems can sustain in the long term without irrigation. Hence, there is a need to increase the understanding of the water balance for rainfed cropping systems in the NCP. However, evapotranspiration (ET), i.e., the sum of soil evaporation (E) and plant transpiration (T), is often reported as one component because the measurement of E and T separately in the field is difficult and costly^31^,^32^,^33^. Alternatively, crop models can be used to estimate E and T when properly calibrated and validated, including the FAO AquaCrop model^34^,^35^,^36^,^37^. Data of long-term field experiments can provide a robust basis for further improving the calibration and validation of models.

Here, we report on a comprehensive and quantitative analysis of data from a long-term (30-years) field trial, which was combined with a model simulation study. No results of this trial have been published before, apart from an analysis of soil organic matter changes as function of fertilizer and manure treatment^38^. The objectives of our study are: (i) to quantify the effects of long-term fertilization and soil organic amendments on crop yield over time; (ii) to examine rainfall and treatment interactions in crop yield and (iii) to explore options to increase crop yield in rainfed wheat-soybean double cropping system in NCP.

## Materials and Methods

### Site and soil description

**Figure 1:**
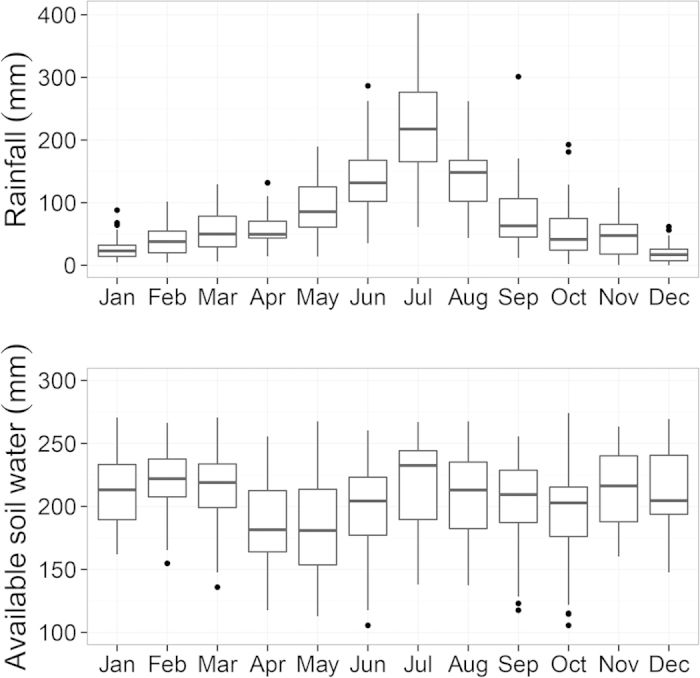
Distribution of monthly rainfall and available soil water in the top 2 meter during the period 1982–2012. Available soil water is defined as the total soil water content minus the water content at wilting point. Boxes show the range between 25^th^ and 75^th^ percentile values, i.e. interquartile between Q1 (25^th^ percentile) and Q3 (75^th^ percentile). Lines in the boxes show the median values. Whiskers show the range of Q1 − 1.5 interquartile at the bottom, and Q3 + 1.5 interquartile at the top. Dots are the outliers beyond the range of Q1 − 1.5 interquartile and Q3 + 1.5 interquartile.

The long-term field experiment is located at Madian Agro-Ecological Station in the North China Plain (N33°13′, E116°37′). The area has a sub-humid climate, with mean annual temperature of 16.5 °C (min. −7.4 °C and max. 36.5 °C). Annual precipitation ranged from 400 to 1500 mm during the last 30 years, about 70% of which occurs from May to September (Fig. 1).

The site is flat (slope < 1%) and has been cultivated for many years. The predominant Vertisols have developed in fluvial and lacustrine deposits. They are classified as Calcic Kastanozems, according to the soil classification system of the Food and Agriculture Organization (FAO). Soil pH ranges from 6.0 to 8.6 and soil organic carbon (SOC) content ranges from 5.8 to 7.5 g kg^−1^. Main topsoil (0–20 cm) characteristics in the experimental field at the start in 1982 were as follows: soil bulk density: 1.45 g cm^−3^, pH: 7.4, sand (0.2 to 0.02 mm): 280 g kg^−1^, silt (0.02 to 0.002 mm): 306 g kg^−1^, clay (<0.002 mm): 414 g kg^−1^, SOC content: 5.8 ± 0.08 g kg^−1^, total N content: 0.96 ± 0.04 g kg^−1^, total P content: 0.28 ± 0.02 g kg^−1^. Information on soil bulk density, field capacity and wilting point for different layers up to 200 cm are shown in Table 1.

### Cropping practice and experimental design

A winter wheat-soybean rotation is common practice in the region. At Madian Agro-Ecological Station, winter wheat (*Triticum aestivum L.*), mainly variety Yedan 13, was grown from late October to May, and soybean (*Glycine max*), mainly variety Zhonghuang 13, from June to September.

The long-term field experiment was initiated in 1982, and had six treatments (Table 2): no fertilization (CK), mineral NPK fertilizer alone (T1), mineral fertilizer combined with 2.5 ton ha^−1^ yr^−1^ of wheat straw (T2), mineral fertilizer combined with 5 ton ha^−1^ yr^−1^ of wheat straw (T3), mineral fertilizer combined with 7.8 ton ha^−1^ yr^−1^ of pig manure (T4), and mineral fertilizer combined with 12.5 ton ha^−1^ yr^−1^ of cattle manure (T5). The treatments were laid out in a randomized block design with four replications.

The plot size was 70 m^2^ (14.9 m × 4.7 m). All plots were ploughed (0–20 cm) after each harvest. Mineral N, P and K fertilization was applied as urea, calcium superphosphate and potassium chloride, respectively. The amounts of fertilizers applied were similar to the recommended amounts for this cropping system, i.e., 180 kg N, 90 kg P_2_O_5_, and 135 kg K_2_O per ha per year. All fertilizers were applied as base fertilizer at once at the start of the wheat growing season in October. No fertilizers were applied to soybean.

Herbicides and pesticides were applied when necessary. Wheat and soybean were harvested manually and all above-ground biomass were removed from the experimental plots, except for the stubble. Grains yields were air dried, threshed and then weighted.

### Soil and plant sampling and analyses

Soil samples were collected from the top 20 cm after the soybean harvest in October of each year. Soil samples were randomly taken from three locations in each plot with a soil core sampler (inner diameter 7 cm) and analysed per plot. The samples were air dried and passed through an 8 mm sieve. Visible pieces of crop residues and roots were removed. The dried and sieved soil was stored in glass jars until analysis. Soil bulk density was measured using the core method and soil pH was measured by the potentiometric method in a soil-water extract (2.5:1, w/v water)^39^. Total N was determined by the method described by Walkley and Black^40^, and total P by Murphy and Riley^41^. Available N was measured by the alkali N-proliferation method, available P (Olsen-P) was extracted by 0.5 mol L^−1^ NaHCO_3_ (pH 8.5) and then measured via Mo-Sb colorimetric method, available potassium was extracted by 1 mol L^−1^ NH_4_-OAc (pH = 7) with 1:5 ratio of weight:volume, and then measured via flame photometer method^39^. Soil water contents were measured every 10 days by using the gravity method. The top 50 cm was sampled in 10 cm intervals, from 50 to 100 cm at 25 cm intervals and from 100 to 200 cm at 50 cm intervals. Each sample consisted of 5 subsamples, taken randomly on fertilized strips adjacent to the experimental plots (to avoid damage through frequent sampling of the experimental plots). Plant N, P and K contents were analysed at harvest once in every five years. Plant N content was measured by Kjeldahl method. Plant P content was measured by the Mo-Sb colorimetric method and plant K content was measured by flame photometer^39^.

### Definitions and calculations

The water balance for each crop growing seasons is defined as follows:


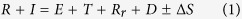


where *R* is rainfall, *I* is irrigation, *E* is soil evaporation, *T* is crop transpiration, *R*_*r*_ is runoff, *D* is drainage and Δ*S* is the change in soil moisture (or soil water content), all with the unit mm.

Irrigation was not applied in this study and runoff rarely occurred (the plots were built with bunds to avoid runoff), and therefore was neglected. Hence, the water balance was simplified to:


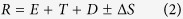


Water use efficiency (WUE, in kg m^−3^) is defined as:


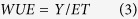


where *Y* is grain yield (kg ha^−1^), *ET* is evapotranspiration (mm), i.e. the sum of *E* and *T* during each crop growing season.

Nitrogen use efficiency (NUE, in kg kg^−1^) is defined as agronomic efficiency of applied N^42^:


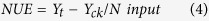


where *Y*_*t*_ is yield of fertilization treatment (kg ha^−1^), *Y*_*ck*_ is yield of CK treatment.

Similarly, phosphorus use efficiency (PUE) and potassium use efficiency (KUE) (in kg kg^−1^) are defined as:


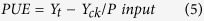



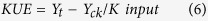


Please note that NUE, PUE and NUE were determined for wheat only because soybean did not receive any NPK fertilization.

### Descriptions of the AquaCrop model

The FAO AquaCrop model is a water-driven crop growth model, which can simulate crop biomass and yield as function of climate and water availability^43^,^44^. AquaCrop requires 4 main sets of input data, i.e. (1) climate data (rainfall, minimum and maximum temperature and reference evapotranspiration (ET_0_)), (2) crop parameters, (3) soil data and (4) field management data. Climate data (e.g., rainfall, minimum and maximum temperature) were collected from a near-by meteorological station (200 meters away from the experimental site). ET_0_ was calculated by the FAO Penman-Monteith equation as described in Allen, *et al.*^45^. Soil data and field management data were derived from measurements. The AquaCrop model was calibrated and validated previously in Qin, *et al.*^34^. A full list of crop parameters used in this study is summarized in Table S1. The calibration and validation procedures and evaluations of the model performance are documented in greater detail in Qin, *et al.*^34^.

### Model experiments

To investigate the mean effects of straw mulching, plastic cover and irrigation on crop yields for the period of 1982–2012, we set up 4 model experiments (E1–E4) as follows:


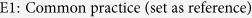



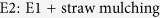



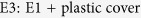



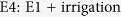


Model experiment E1 simulated crop growth with two levels of initial soil water content at seeding, i.e. a low level with 60% field capacity (FC) and a high level with 75% FC. The range from 60 to 75% FC largely represented the initial soil water content during the experimental period. Model experiment E2 aimed at testing the effects of straw mulching, and model experiment E3 for the effects of plastic film cover. The effectiveness of straw mulching in reducing soil evaporation was estimated as 50% and that of plastic cover at 90%, which are the default values in the AquaCrop model^44^. However, plastic film covers around 80% of the field in practice. Hence, the overall soil evaporation reduction by plastic film was set at 72%. Model experiments E4 tested the effects of a smart irrigation that automatically replenishes the soil water content back to field capacity when 80% of total available soil water is depleted during the crop growing season. We ran these model experiments for the period of 1982–2012, assuming no soil fertility stress and keeping all input data (e.g., climatic and soil information) and crop parameters the same in all simulations.

### Statistical analyses

Data were analysed with a mixed-effect model via R package “Lme4”^46^,^47^. A mixed-effect model is a statistical model containing both fixed effects and random effects. We quantified the effects of main variables, i.e. (1) rainfall, (2) treatments (CK, T1–T5), (3) rainfall and treatment interactions on crop yields as:


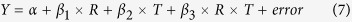


where *Y* is yield (ton ha^−1^), α is the intercept (ton ha^−1^), R is rainfall (mm), *β*_1_ is the effect of rainfall (fixed variable 1), *β*_2_ is the effect of a specific treatment (fixed variable 2), *β*_3_ is the effect of rainfall and treatment interactions, and *error* represents the residual effects that were not taken into consideration. Trial years are considered as random variable in the model.

An additional analysis considering the interactions between rainfall and N, P and K input on yields was conducted as follows:


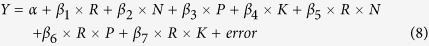


where *β*_2–4_ represent the effect of N, P and K, and *β*_5–7_ the effect of interactions between rainfall and N, P and K respectively. N, P and K input were the sum of that in fertilizer, manure and straw.

## Results

### Rainfall and soil water content

Long-term average annual rainfall (1981–2012) at the station was ~900 mm, and heavily influenced by the monsoon. Around two-third of the annual rain (~600 mm) fell in June to September, which is the growing season for soybean (Fig. 1), and the other third (~300 mm) fell during the winter wheat growing season from October to May. High-yielding winter wheat and soybean crops normally require 450 to 650 mm of water, depending on climate, yield and length of the growing period and monthly distribution^48^. Evidently, winter wheat received relatively little rainwater, compared to soybean.

Soil moisture contents decreased during the wheat growing season and increased during the soybean growing season. Available soil water (ASW) was defined as the measured soil moisture content minus the moisture content at wilting point in a 2 meter’s profile. Mean ASW was lowest in May when wheat was harvested (Fig. 1). From June, soil water content started to increase again. Winter wheat was planted in October, but soil water content did not change much till February because of relatively low temperature and slow development of wheat in the early stage. From March, soil water started to decrease, because evapotranspiration (ET) exceeded rainfall. On average, the upper 2 m of soil contained around 200 mm of available water, ranging from 100 to 280 mm. The top 1 m of the soil profile accounted for ~70% of the changes in soil moisture content in the whole soil profile of 2 m.

### Crop yields

**Figure 2:**
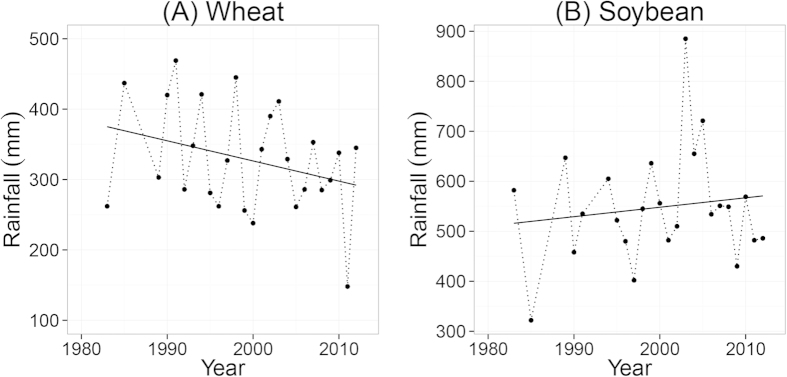
Total rainfall of the growing seasons of wheat (**A**) and soybean (**B**) during the period 1982–2012. Dots represent the amount of rainfall (mm) and solid lines show the trends.

Figure 2 shows the ranges of wheat and soybean yields as function of fertilization treatments. Yields were low in the CK treatment. Fertilization and soil organic amendments significantly increased yields. The ranking of the treatments, from high to low yields, was T5 ≥ T4 > T3 ≥ T2 ≥ T1 ≫ CK. Mean wheat yield in CK was only 1 ton ha^−1^, which is ~20% of the mean yields in the fertilized treatments (Fig. 2A). Mean soybean yield in CK was 0.8 ton ha^−1^, which is ~40% of the mean yields in the fertilized treatments (Fig. 2B).

The NPK + manure treatments (T4 and T5) significantly increased wheat yields relative to NPK alone (T1). Treatments NPK + straw (T2 and T3) did not significantly increase wheat yields compared to NPK alone (Table 3). The results of soybean were rather similar to that of wheat, but NPK + high rate straw (T3) also significantly increased soybean yields relative to the NPK alone treatment.

Fertilization and soil organic amendments also increased yield stability over time (Table 4). The mean coefficient of variance (CV) in CK was 0.39 for both crops, while mean CV in the fertilization and soil organic amendment treatments ranged from 0.12–0.15 for wheat and from 0.12–0.18 for soybean.

**Figure 3:**
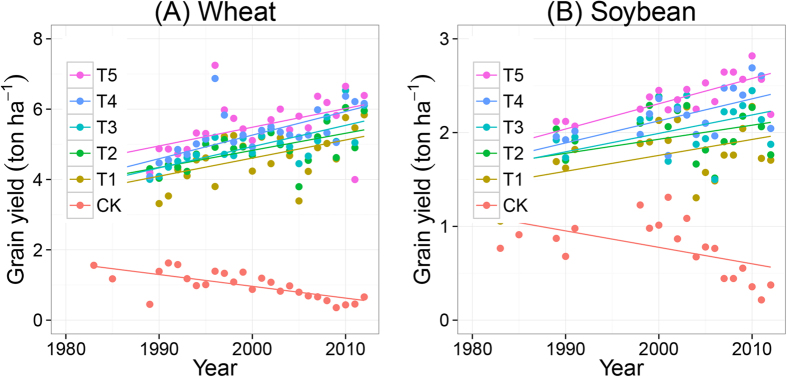
Trends of wheat (**A**) and soybean (**B**) yields over time during the period 1982–2012, as function of fertilization treatments. The treatments are noted as: no fertilization (CK), mineral NPK (T1), NPK + low rate of straw (T2), NPK + high rate of straw (T3), NPK + pig manure (T4) and NPK + cattle manure (T5). Statistics are summarized in Table 4.

Wheat and soybean yields increased over time in fertilized treatments T1 to T5, whereas the opposite occurred in the CK treatment (Fig. 3). Wheat yields in fertilized treatments T1–T5 increased on average by 50–70 kg ha^−1^yr^−1^ and soybean yields increased on average by 10–30 kg ha^−1^yr^−1^ (Table 4). Over the 30 years’ time of the trial, wheat and soybean yield increased by 1.5–2.1 and 0.3–0.9 ton ha^−1^ in the fertilized treatments, whereas yields decreased on average by ~0.9 and ~0.6 ton ha^−1^ in the CK treatment for wheat and soybean, respectively.

### Soil fertility

**Figure 4:**
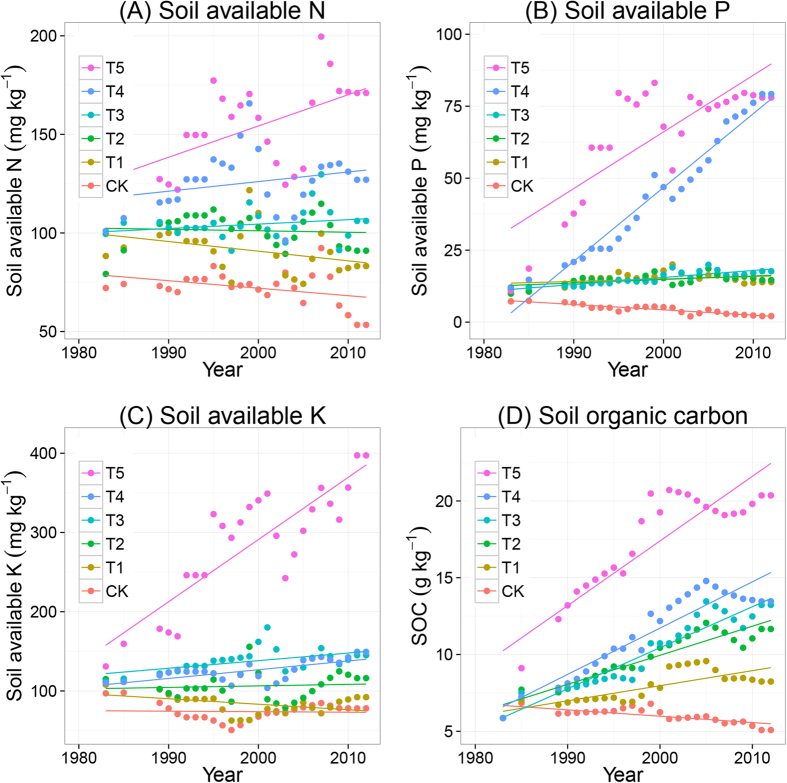
Trends of soil available (mineral) N (**A**), soil available (Olsen) P (**B**), soil available (Exchangeable) K (**C**), and soil organic carbon (**D**), over time during the period 1982–2012, as function of fertilization treatments. The treatments are noted as: no fertilization (CK), mineral NPK (T1), NPK + low rate of straw (T2), NPK + high rate of straw (T3), NPK + pig manure (T4) and NPK + cattle manure (T5). Note that Olsen P levelled off at ~75 mg kg^−1^ and SOC levelled off at 20 g kg^−1^.

Long-term fertilization increased soil fertility characteristics and SOC content over time (Fig. 4). Over the 30 years’ period, NPK + cattle manure (T5) increased soil available N, P and K and SOC content most among all treatments. Soil available N at harvest time of soybean ranged from 125 to 175 mg kg^−1^ in the NPK + cattle manure treatment (T5), and from 100 to 125 mg kg^−1^ in the NPK + pig manure treatment (T4). Soil available N was around 75 to 125 mg kg^−1^ in the NPK alone (T1) and NPK + straw treatments (T2 and T3), and ranged from 50 to 75 mg kg^−1^ in the CK treatment. Olsen P increased from 12.5 to about 75 mg kg^−1^ in the NPK + manure treatments (T4 and T5), increased only very slightly in the NPK alone and NPK + straw treatments (T2, T3), and dropped to about 5 mg kg^−1^ in the CK treatment. Cattle manure application significantly increased soil available K much stronger than pig manure and straw return. Soil available K slightly decreased in the NPK alone and CK treatments, suggesting that the annual application of 135 kg K_2_O ha^−1^ yr^−1^ was not sufficient to compensate for the annual K withdrawal in harvested crop and leaching losses. Soil organic carbon (SOC) content increased in all fertilized treatments but decrease in CK over time. SOC levelled off at ~20 g kg^−1^ in the treatment with cattle manure addition, and at 10–15 g kg^−1^ in the treatments with pig manure and straw. Fertilizer NPK alone slightly increased SOC content during the 30 years’ period (Fig. 4).

### Interactions between rainfall and fertilization in yields

The effects of rainfall, fertilization (T1–T5) and their interactions in yields are shown in Table 5. The results of the treatments T2, T3, T4 and T5 were compared to the yields in the NPK alone treatment (T1), as reference. We excluded the results of the CK treatment in the statistical analysis for two reasons: first, to reduce the variance in the dataset caused by the CK treatment; and second, to examine the differences between the treatments with chemical NPK fertilizers alone and those with NPK fertilizers plus manure or straw. There were positive interactions between rainfall and NPK + manure treatments (T4 and T5) in wheat yields; the interactive effects were larger with cattle manure than with pig manure. These positive interactions suggest that years with relatively high rainfall increased wheat yields in treatments with NPK + manure, compared to treatments with NPK alone and NPK + straw (Table 5).

There were no significant interactions between rainfall and fertilization in soybean yields (Table 5), most likely because rainfall was abundant during the soybean growing season. Though soybean did not receive any fertilization, yields clearly benefited from the residual effects of the fertilizers, manure and straw applied to the wheat crop (Fig. 2).

An additional analysis indicated that there were significant positive interactions between rainfall and K input in wheat yields, suggesting that the wheat benefited from the additional K inputs via straw and manure during years with relatively high rainfall (Table S2). Wheat yields in treatments T1–T5 were not related to total N and P inputs, suggesting that the N and P inputs via NPK fertilizers alone were sufficient, and the N and P inputs via straw and manure were redundant. In other words, the NPK inputs via manures can partially replace fertilizer NPK inputs.

### Water balance and the portioning of E and T in wheat and soybean growing seasons

**Figure 5:**
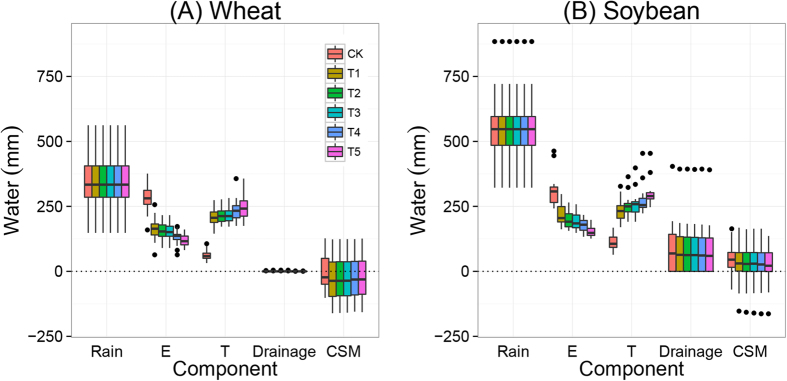
Water balance components for the wheat (**A**) and soybean (**B**) growing seasons during the period 1982–2012 as function of fertilization treatments: no fertilization (CK), mineral NPK (T1), NPK + low rate of straw (T2), NPK + high rate of straw (T3), NPK + pig manure (T4) and NPK + cattle manure (T5). Boxes show 25^th^ and 75^th^ percentiles (i.e. Q1 and Q3). Lines in the boxes show the median values. The components of water balance are shown on the x-axis, i.e., rainfall, soil evaporation (E), crop transpiration (T), drainage and changes in soil moisture (CSM). See formula (2) and text for explanations.

Figure 5 shows the water balance of the wheat and soybean growing seasons during the period 1982–2012, estimated by the AquaCrop model. The partitioning between soil evaporation (E) and crop transpiration (T) differed between treatments. Evaporation (E) was relatively high in the CK treatments, and transpiration (T) was relatively high in the fertilization treatments. During the wheat growing season, mean E was 280 mm and mean T was 60 mm in the CK treatment, i.e., T/ET ratio was 18%, while mean E was 120 and mean T 240 mm in the NPK + cattle manure treatment (T5), i.e., T/ET ratios was 67% (Fig. 5A). In the soybean growing season, mean E was 310 mm and mean T 110 mm in the CK treatment, i.e., T/ET ratio was 26%, while mean E was 150 and mean T 300 mm in the NPK + cattle manure treatment (T5), i.e., T/ET ratios was 67%. Hence, the T/ET ratio greatly increased through fertilization.

### Water and nutrient use efficiencies

**Figure 6:**
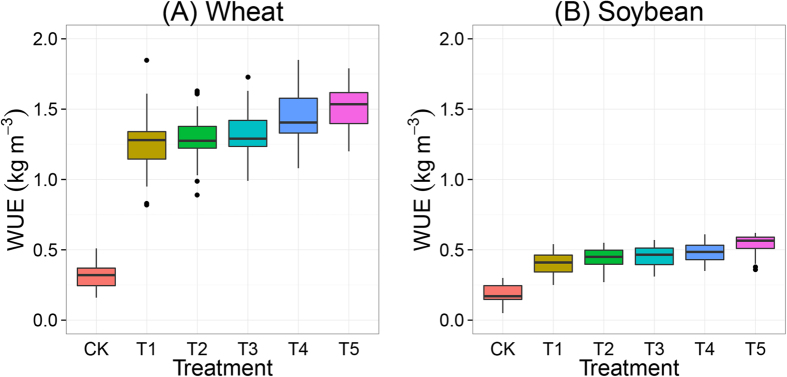
Water use efficiency (WUE) of wheat (**A**) and soybean (**B**) as function of fertilization treatments during the period 1982–2012. The treatments are noted as: no fertilization (CK), mineral NPK (T1), NPK + low rate of straw (T2), NPK + high rate of straw (T3), NPK + pig manure (T4) and NPK + cattle manure (T5). Boxes show 25^th^ and 75^th^ percentiles (i.e. Q1 and Q3). Lines in the boxes show the median values.

Water use efficiency (WUE) was strongly related to yield (Fig. 6). The ranking of treatments, from high to low WUE, was T5 > T4 > T3 > T2 > T1 > CK. Mean WUE of wheat was 0.3 kg m^−3^ in CK, and ranged from 1.3 to 1.7 kg m^−3^ in the fertilized treatments. Mean WUE of soybean was 0.2 kg m^−3^ in CK, and ranged from 0.4 to 0.6 kg m^−3^ in the fertilized treatments.

**Figure 7:**
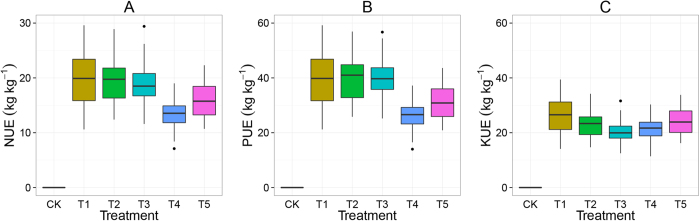
Effects of fertilization and soil conservation treatments on nitrogen use efficiency (**A**), phosphorus use efficiency (**B**) and potassium use efficiency (**C**) in wheat, as function of fertilization treatments during the period 1982–2012. The treatments are noted as: no fertilization (CK), mineral NPK (T1), NPK + low rate of straw (T2), NPK + high rate of straw (T3), NPK + pig manure (T4) and NPK + cattle manure (T5). Boxes show 25^th^ and 75^th^ percentiles (i.e. Q1 and Q3). Lines in the boxes show the median values. Please note that the values for CK were not zero but not applicable because there were no NPK input in CK.

Figure 7 presents the apparent nutrient use efficiencies of wheat. Note that the nutrient recovery by soybean was disregarded, although soybean benefitted from the residual effects of NPK fertilization and the soil organic amendments (Figs 2 and 3). Also, N, P and K use efficiencies are confounded, because they were applied in combination as NPK fertilizers, straw and manures. As a result, the nutrient use efficiencies presented here for wheat are underestimates and therefore indicated as ‘apparent efficiencies’. The mean apparent N use efficiency (aNUE) of wheat ranged from 13–20 kg kg^−1^. There were no significant differences in aNUE between treatment NPK alone (T1) and NPK + straw (T2 and T3), but NPK + manure had a significantly lower aNUE than treatment NPK alone. Mean aPUE ranged from 25–35 kg kg^−1^; there were no significant differences between NPK fertilization alone (T1) and NPK fertilization + straw (T2 and T3) in aPUE. Animal manures provided extra P input (50–70 kg ha^−1^ yr^−1^) and led to a lower aPUE in treatment T4 and T5 compared to T1. Mean aKUE ranged from 20–26 kg kg^−1^. NPK fertilization (T1) resulted in the highest aKUE. Adding straw and animal manures led to lower aKUE in T2–T5 relative to T1 because of the added K via straw and manures.

### Effects of straw mulching, plastic and irrigation

**Figure 8:**
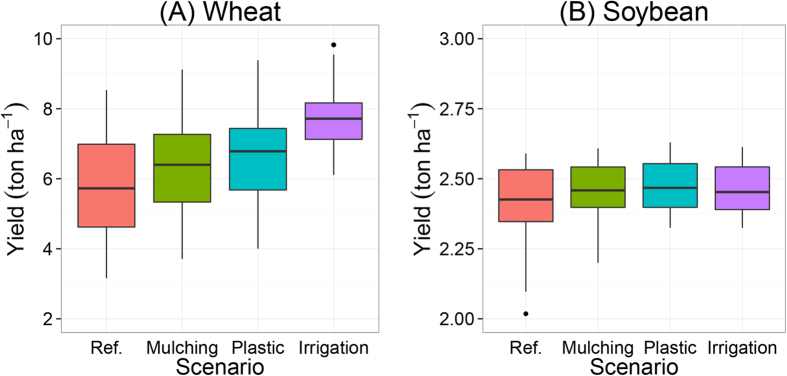
Simulated wheat (**A**) and soybean (**B**) yields with straw mulching, plastic film cover and irrigation for the period 1982–2012. The reference (Ref.) is the simulated yield with common range of initial soil water content of 60–75% field capacity.

**Figure 9:**
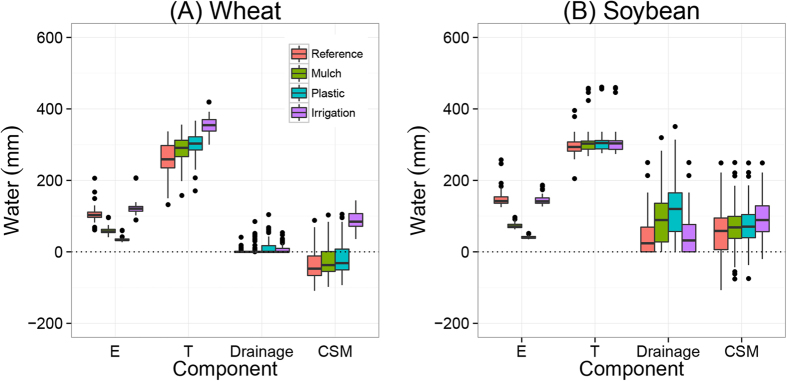
Simulated components of the water balances for wheat (**A**) and soybean (**B**) as function of straw mulching, plastic film cover and irrigation.

Figure 8 shows the calculated effects of straw mulching, plastic film cover and irrigation on wheat and soybean yields. Straw mulching and plastic cover may increase mean wheat yields by 10–15% and irrigation by 35%, compared with the reference yields (Fig. 8A). Straw mulching, plastic cover and irrigation had no effects on soybean yields, because soybean yields were not limited by water (Fig. 8B). Mulching and plastic cover increased WUE of wheat by 15–25%, supplementary irrigation with 100 mm of water increased WUE by 5% (Fig. 9A). Straw mulching, plastic film cover and irrigation decreased the variation in yield between years. Effects of mulching, covers and irrigation were larger in relatively dry years than in relatively wet years.

## Discussion

### Imbalances in fertilization

Wheat and soybean yields in the unfertilized control treatment (CK) of our long-term field experiment were strongly limited by nutrients. NPK fertilization increased yields of wheat and soybean on average by a factor of 5 and 2.5, respectively, compared to the CK treatment (Fig. 2). NPK + manure increased yields more than NPK + straw and NPK alone. The additional effect of animal manure (compared to NPK alone) is likely due to the increased amounts of available K. Clearly, the recommended rate of 135 kg K_2_O ha^−1^ yr^−1^ was insufficient to replenish the annual K withdrawal in harvested crop and K leaching losses. When assuming a mean K content of 5 g per kg in grain and 10 g per kg in straw (Figure S1), and a harvest index of 45%, the annual mean withdrawal with the harvested wheat crop is 85 kg K ha^−1^ yr^−1^, equivalent to 100 kg K_2_O ha^−1^ yr^−1^ (range 80–120 kg K_2_O ha^−1^ yr^−1^). Withdrawal of K with harvested soybean is estimated at 30–50 kg K_2_O ha^−1^ yr^−1^ in the fertilized treatments. This simple balance calculation indicates that the total K withdrawal with harvested crops is equal to or exceeds the application rate with the chemical NPK fertilizers, and therefore is insufficient for sustaining high yields in the long term, especially when the soil has a relatively low K supplying capacity^25^,^49^.

Potassium deficiency was identified as a constraint to increasing rice yields in Asia in the 1990 s. The low rates of K fertilization practiced at that time were insufficient to replenish the amount of K removed by intensive lowland rice production^50^,^51^. The occurrence of K deficiency is in part also related to the decreasing trend of using manures to cereal crops. China has a long history of using animal manures as nutrient input to crops. However, the application of organic manures to cereal crops dramatically decreased since the 1980 s. As a result, K deficiency also became a limiting factor for rice, wheat and maize production in many regions in China^25^,^49^. In our study, manure application provided extra K input of some 50–60 kg ha^−1^, which likely contributed to increased wheat yields.

Manure application contributed to P accumulation in the soil; Olsen P values in the treatment NPK fertilizer + manure (T4 and T5) rapidly increased to around 75 mg kg^−1^ and then remained at this level during the course of the experiment, suggesting soil P saturation and leaching of P from the top soil to the subsoil. The optimal P Olsen level for cereals is in the range of 10 to 20 mg kg^−1^ 52. Olsen P remained within the recommended range of 10–20 mg kg^−1^ in the treatments T1–T3, suggesting that the P inputs via NPK fertilizers and straw were adequate. Manure application also provided extra N of 100–130 kg ha^−1^. However, wheat yields in the fertilized treatments were not related to N input (Table S2), suggesting that the N inputs via NPK fertilizers and biological N_2_ fixation by soybean were adequate.

Soybean did not receive fertilization in the long-term experiment but clearly benefited from the residual effects of the chemical fertilizers, straw and manures applied to wheat in autumn (Figs 2 and 3). A meta-analysis of soybean N uptake and N fixation showed that, on average, ~55% of soybean N demand was met by biological N_2_ fixation^17^. In most cases, the amount of N fixed was not sufficient to produce high soybean yield (4–5 ton ha^−1^). The partial N balance (fixed N in aboveground biomass—N in seeds) was negative in 80% of all data sets, with a mean net soil N mining of 40 kg N ha^−1^ 17. There was a slightly decreasing trend in soil available N in treatment T1 (NPK fertilizer alone) and a slightly increasing trend in SOC content (Fig. 4) clearly indicating that the supply of N in this treatment was not excessive. When assuming a mean N content of 15 g per kg in grain and 5 g per kg in straw (Fig S5), and a harvest index of 45%, the annual mean withdrawal with the harvested wheat crop is 105 kg N ha^−1^ yr^−1^ (range 80–125 kg N ha^−1^ yr^−1^). Withdrawal of N with harvested soybean is estimated at 55 kg N ha^−1^ yr^−1^ (range 25–75 kg N ha^−1^ yr^−1^) in the fertilized treatments. This indicates that the total N withdrawal with harvested crops was close to the N application rate of 180 kg ha^−1^ yr^−1^, especially during the second half of the experimental period when grain yields and N contents were slightly higher compared to the first half of the experimental period (Figure S1).

### Water deficiency and fertilization effects

High-yielding wheat varieties require 450 to 650 mm of water, depending on climate, yield and length of the growing period^48^. In this study, the estimated ET of wheat ranged from 210 to 500 mm (mean = 360 mm), and that of soybean from 370 to 630 (mean = 440 mm). Likely, wheat yields in the fertilized treatments were limited by low seasonal rainfall (mean = 300 mm). The estimated mean ET was comparable with previous studies on wheat-soybean double cropping systems. For example, Daniels and Scott^53^ reported that the mean ET of rainfed wheat was 328 mm. For irrigated and non-irrigated soybeans, the mean ET was 375 and 255 mm, respectively. Caviglia, *et al.*^54^ reported that mean ET of wheat ranged from 313 to 334 mm, and that of soybean from 359 to 434 mm. Singh, *et al.*^55^ reported that mean ET of wheat and soybean were around 350 and 400 mm, respectively.

Nutrient availability greatly affected crop transpiration and thereby yields; mean T/ET ratio was low in the CK treatments, i.e. 18% for wheat and 27% for soybean. Low T (and thereby low T/ET ratio) in the CK treatments was mainly due to severe soil nutrient depletion (Fig. 5). Olsen P dropped to values of <5 mg kg^−1^, and soil available N and K also had decreasing trends in CK (Fig. 4). Nutrient deficiency in CK significantly limited crop growth and thereby led to low transpiration and relatively large soil evaporation. In the fertilized treatments, the T/ET ratio was 56–67% for wheat and 52–66% for soybean (Fig. 5). Similar T/ET ratios (60–70%) have been reported for wheat by some previous studies^56^,^57^,^58^. Despite the large differences in T/ET ratios between fertilized and unfertilized treatments, the total amount of water consumed as evapotranspiration (ET) was rather similar (differences <30 mm), indicating that ET was not strongly affected by fertilization, as observed also by some previous studies^59^,^60^,^61^. Therefore, yields were often more closely related to transpiration than to total available water (Figure S2).

Compared to semi-arid regions in Northwest China, Anhui has relatively high annual rainfall (900 mm), which provides the possibility to grow two crops per year. However, the annual yields of the rainfed wheat-soybean system highly depend on the amount of rain. Soil mulching with straw or plastic are common measures to reduce evaporation and thereby increasing crop yields and WUE in Northwest China^62^,^63^, but not in Anhui. Our model simulations indicate that straw mulching and plastic cover could increase mean wheat yield by 10–15%, and supplementary irrigation of 100 mm could increase mean wheat yields by 35% (Fig. 7A). Most of the straw is currently used as animal feed, burned or ploughed down in the soil. In treatments with chemical NPK fertilizers and straw (T2 and T3), the straw is ploughed into the soil after harvest of the wheat. Further field studies need to be conducted to evaluate the effectiveness, efficiency and feasibility of straw mulching and plastic covers in this region.

### Increasing trend of crop yields

Over the 30 years’ period, mean wheat and soybean yields increased by 1.5–2.1 and 0.3–0.9 ton ha^−1^, respectively, in the fertilization and soil organic amendment treatments. In contrast, yields decreased in the control treatments. Increasing trend of crop yields may be partially related to the new varieties. According to the study of Evenson and Gollin^64^, improvements in breeding accounted for around 50% of the increase in global cereal yields, whereas the other factors, such as fertilizers, irrigation, mechanization, and improved labour skill together accounted for the other 50% of the increase in yield. In this study, we were not able to quantify the contributions of all factors, but improved genetic varieties and improved crop husbandry (including weed and pest control) certainly have played a role. Inputs of fertilizers, straw and manure did not change over time in this study (Table 2).

Long-term fertilization and addition of organic amendments led to increased soil organic matter contents over time^38^, which may also have contributed to increased crop growth and development. Li, *et al.*^65^ reported that long-term additions of animal manure increased soil moisture availability by 30 to 45 mm in a 2 meter deep soil profile. Contents of SOC increased significantly in the treatments with manure and straw (Fig. 4), but we have no experimentally derived data that indicated how much soil moisture availability changed over time. Likely, some of the yield difference between the treatments with chemical fertilizer without and with manure has to be attributed to the increased SOC content and to the likely increased soil moisture availability in the treatments with animal manure. There were clear correlations between SOC content and crop yield (Figure S3), but the relationship between SOC and yield is highly nested to soil available N (soil N mineralization) and K. Further, manure provides also other essential nutrient elements than NPK (including sulphur, copper, zinc), which may have contributed to the yield difference between treatments T1 and T4/T5.

Contrasting findings from long-term field experiments have also been reported in the literature. For example, yield stagnation and also negative yield trends have been observed in continuous rice-based systems, with unbalanced use of inorganic N and NP application. Positive yield trends have been observed in treatments with NPK and NPK + FYM^66^. The causes of yield decline are location specific, but nutrient depletion and soil-borne diseases are common factors^66^. Cai and Qin^67^ pointed out that application of chemical NPK fertilizers was able to sustain high crop yields, but was unable to contribute much to carbon sequestration in soil. Therefore, mixed applications of organic and inorganic fertilizers is the preferred strategy to ensure long-term food security and soil carbon sequestration in the NCP. Our finding are largely in line with other long-term field studies, i.e., crop yield increased over time in treatments with mineral NPK fertilization and in NPK + manure treatments, but decreased in unbalanced fertilization treatments^26^,^66^,^67^,^68^.

## Conclusions

Long-term fertilization significantly improved soil fertility over time, which enhanced crop transpiration and thereby crop yields. On average, wheat yields were 5 times and soybean yields were 2.5 times higher in the fertilized treatments than in the unfertilized control (CK). Among the fertilized treatments, NPK + manure increased yields more than NPK + straw, and also more than NPK alone. The amount of nutrient provided by NPK fertilizers appeared to be sufficient for N and P, but likely not for K. The additional effect of animal manure (compared to NPK alone) is likely due to the increased amounts of available K, and possibly the supply of sulphur and micronutrients. Long-term NPK fertilization and soil organic amendments also significantly improved yield stability of wheat and soybean.

Manure application rapidly increased P-Olsen to ~75 mg kg^−1^. Thereafter P-Olsen did not increase further, suggesting leaching of P from the top soil to the subsoil. Clearly, manure application had additional effects to mineral NPK fertilizers. However, application rates of NPK + manure were too high for optimal fertilization; manure could have partially replaced the mineral NPK fertilizer input.

Model simulations reveal that wheat yields were limited by water. Yields can be increased by 15% through soil mulching or by 35% through irrigation. The effectiveness of these measures needs to be tested further in the field. The productivity and the sustainability of the rainfed double-crop production systems in the North China Plain rely on integrated soil-water-nutrient-crop management in a site and crop specific manner.

## Additional Information

**How to cite this article**: Qin, W. *et al.* Productivity and sustainability of rainfed wheat-soybean system in the North China Plain: results from a long-term experiment and crop modelling. *Sci. Rep.* **5**, 17514; doi: 10.1038/srep17514 (2015).

## Supplementary Material

Supplementary Information

## Figures and Tables

**Table 1:** Soil bulk density, field capacity and wilting point for different soil layers.

Soil layer	Bulk density	Field capacity	Wilting point
cm	g cm^−3^	v v^−1^ in %	v v^−1^ in %
10	1.26	36.8	12.3
20	1.42	42.6	13.8
30	1.47	36.0	14.7
40	1.46	39.3	16.4
50	1.37	36.4	15.3
60	1.33	32.6	13.4
80	1.40	31.1	14.8
100	1.41	31.0	15.9
200	1.41	31.0	15.9

**Table 2:** Application rates of mineral fertilizers, wheat straw and pig and cattle manure as function of treatment (kg ha^−1^yr^−1^).

Treatment	Mineral fertilizers			Organic soil amendments		
	N	P_2_O_5_	K_2_O	Wheat straw	Pig manure	Cattle manure
CK	0	0	0	0	0	0
T1	180	90	135	0	0	0
T2	180	90	135	2500	0	0
T3	180	90	135	5000	0	0
T4	180	90	135	0	7800	0
T5	180	90	135	0	0	12500

**Table 3:** Results of the statistical analyses of wheat and soybean yields.

Crop	Treatment†	Mean	SD	CV	p value	Sign.‡
Wheat	CK	0.988	0.382	0.386	0.000	***
	T1	4.569	0.695	0.152	1.000	NS
	T2	4.790	0.597	0.125	0.225	NS
	T3	4.888	0.640	0.131	0.091	NS
	T4	5.211	0.747	0.143	0.002	**
	T5	5.445	0.793	0.146	0.000	***
Soybean	CK	0.765	0.299	0.391	0.000	***
	T1	1.768	0.325	0.184	1.000	NS
	T2	1.939	0.317	0.164	0.101	NS
	T3	2.004	0.315	0.157	0.025	*
	T4	2.140	0.297	0.139	0.001	***
	T5	2.325	0.284	0.122	0.000	***

The yield of treatment T1 was set as reference for the t-test. SD is standard deviation. CV is coefficient of variation, i.e. SD divided by mean.

^†^Treatments are explained in Table 2.

^‡^Significance were displayed with numbers of asterisk, ‘***’ means p value ≤ 0.001, ‘**’ means 0.001 < p value ≤ 0.01, ‘*’ means 0.01 < p value ≤ 0.05 and ‘NS’ means p value > 0.05, i.e., not significant.

**Table 4:** Results of the statistical analysis of the trends in wheat and soybean yields over time.

Crop	Treatment	Intercept†	Slope	r^2^	p value	Sign.‡
Wheat	CK	1.564	−0.033	0.509	0.000	***
	T1	3.682	0.051	0.363	0.001	**
	T2	3.951	0.049	0.439	0.000	***
	T3	3.847	0.060	0.590	0.000	***
	T4	4.054	0.067	0.535	0.000	***
	T5	4.528	0.053	0.298	0.004	**
Soybean	CK	1.093	−0.018	0.266	0.020	*
	T1	1.451	0.017	0.210	0.042	*
	T2	1.659	0.015	0.172	0.069	NS
	T3	1.637	0.020	0.301	0.012	*
	T4	1.702	0.024	0.482	0.001	***
	T5	1.822	0.027	0.692	0.000	***

^†^The unit of intercept is ton ha^−1^.

^‡^‘***’ means p < 0.001, ‘**’ means 0.001 ≤ p ≤ 0.01, ‘*’ means 0.01 < p < 0.05 and ‘NS’ means p ≥ 0.05, i.e., not significant.

**Table 5:** Results of the statistical analysis of the effects of rainfall (R), fertilization treatments (T1 to T5 and CK; see Table 2), and their interactions in yields of wheat and soybean.

Crop	Item†	Estimate	Std. Error	df	t value	p value	Sign.
Wheat	(Intercept)	5.310	0.537	42.140	9.886	0.000	***
	β_1_ (R)	−0.002	0.002	42.140	−1.423	0.162	NS
	β_2_ (T2)	−0.426	0.401	103.970	−1.064	0.290	NS
	β_2_ (T3)	−0.169	0.401	103.970	−0.421	0.674	NS
	β_2_ (T4)	−0.254	0.401	103.970	−0.634	0.527	NS
	β_2_ (T5)	−0.990	0.401	103.970	−2.472	0.015	*
	β_3_ (R*T2)	0.002	0.001	103.970	1.665	0.099	NS
	β_3_ (R*T3)	0.001	0.001	103.970	1.256	0.212	NS
	β_3_ (R*T4)	0.003	0.001	103.970	2.306	0.023	*
	β_3_ (R*T5)	0.005	0.001	103.970	4.802	0.000	***
Soybean	(Intercept)	1.506	0.255	96.480	5.916	0.000	***
	β_1_ (R)	0.000	0.000	95.130	0.916	0.362	NS
	β_2_ (T2)	0.154	0.334	94.250	0.462	0.645	NS
	β_2_ (T3)	0.137	0.334	94.250	0.410	0.683	NS
	β_2_ (T4)	0.696	0.334	94.250	2.081	0.040	*
	β_2_ (T5)	0.620	0.334	94.250	1.856	0.067	NS
	β_3_ (R*T2)	0.000	0.001	94.250	0.050	0.961	NS
	β_3_ (R*T3)	0.000	0.001	94.250	0.303	0.762	NS
	β_3_ (R*T4)	−0.001	0.001	94.250	−0.988	0.326	NS
	β_3_ (R*T5)	0.000	0.001	94.250	−0.193	0.847	NS

^†^Yields of the NPK treatment (T1) are set as the reference. Intercepts show the mean yields of T1.

^‡^Significance were displayed with numbers of asterisk, ‘***’ means p value ≤ 0.001, ‘**’ means 0.001 < p value ≤ 0.01, ‘*’ means 0.01 < p value ≤ 0.05 and ‘NS’ means p value > 0.05, i.e., not significant.

## References

[b1] UNESCO. The United Nations World Water Development Report 3: Water in a Changing World. Paris: UNESCO, and London: Earthscan (2009).

[b2] Rockstrom J. *et al.* Managing water in rainfed agriculture-The need for a paradigm shift. Agr Water Manage 97, 543–550, 10.1016/j.agwat.2009.09.009 (2010).

[b3] Mueller N. D. *et al.* Closing yield gaps through nutrient and water management. Nature. 10.1038/nature11420 (2012). 22932270

[b4] Rockstrom J., Lannerstad M. & Falkenmark M. Assessing the water challenge of a new green revolution in developing countries. P Natl Acad Sci USA 104, 6253–6260, 10.1073/pnas.0605737104 (2007). PMC1851042 17404216

[b5] FAO. How to Feed the World in 2050—High-Level Expert Forum http://www.fao.org/wsfs/forum2050/wsfs-forum/en/(Date of access:05/03/2015). (2009).

[b6] Hoekstra A. Y. & Mekonnen M. M. The water footprint of humanity. P Natl Acad Sci USA 109, 3232–3237, 10.1073/pnas.1109936109 (2012). PMC3295316 22331890

[b7] Rosegrant M. W. & Cline S. A. Global food security: Challenges and policies. Science 302, 1917–1919 (2003). 14671289 10.1126/science.1092958

[b8] Green T. R., Yu Q. A., Ma L. W. & Wang T. D. Crop water use efficiency at multiple scales Preface. Agr Water Manage 97, 1099–1101, 10.1016/j.agwat.2010.03.018 (2010).

[b9] Piao S. L. *et al.* The impacts of climate change on water resources and agriculture in China. Nature 467, 43–51, 10.1038/Nature09364 (2010). 20811450

[b10] de Wit C. T. Transpiration and crop yields. Verslag Landbouwkundig Onderzoek, Wageningen, Netherlands: Wageningen University (1958).

[b11] Marschner H. Mineral Nutrition of Higher Plants. Academic Press, San Diego, 889 pp. (1995).

[b12] Bernacchi C. J. & VanLoocke A. Terrestrial Ecosystems in a Changing Environment: A Dominant Role for Water. Annual Review of Plant Biology. 10.1146/annurev-arplant-043014-114834 (2015). 25621516

[b13] Downton W. J. S., Loveys B. R. & Grant W. J. R. Non-Uniform Stomatal Closure Induced by Water-Stress Causes Putative Non-Stomatal Inhibition of Photosynthesis. New Phytologist 110, 503–509 (1988). 10.1111/j.1469-8137.1988.tb04161.x 33873929

[b14] Chaves M. M. *et al.* How plants cope with water stress in the field. Photosynthesis and growth. Annals of Botany 89, 907–916, 10.1093/Aob/Mcf105 (2002). 12102516 PMC4233809

[b15] Downton W. J. S., Loveys B. R. & Grant W. J. R. Stomatal Closure Fully Accounts for the Inhibition of Photosynthesis by Abscisic-Acid. New Phytologist 108, 263–266 (1988). 10.1111/j.1469-8137.1988.tb04161.x 33873929

[b16] Setiyono T. D., Walters D. T., Cassman K. G., Witt C. & Dobermann A. Estimating maize nutrient uptake requirements. Field Crop Res 118, 158–168, 10.1016/j.fcr.2010.05.006 (2010).

[b17] Salvagiotti F. *et al.* Nitrogen uptake, fixation and response to fertilizer N in soybeans: A review. Field Crop Res 108, 1–13, 10.1016/j.fcr.2008.03.001 (2008).

[b18] He Y., Hou L. L., Wang H., Hu K. & McConkey B. A modelling approach to evaluate the long-term effect of soil texture on spring wheat productivity under a rain-fed condition. Sci Rep 4, Artn 5736 10.1038/Srep05736 (2014). PMC4115211 25074796

[b19] Seufert V., Ramankutty N. & Foley J. A. Comparing the yields of organic and conventional agriculture. Nature 485, 229–U113, 10.1038/Nature11069 (2012). 22535250

[b20] Rigby D. & Caceres D. Organic farming and the sustainability of agricultural systems. Agr Syst 68, 21–40, 10.1016/S0308-521x(00)00060-3 (2001).

[b21] Liu Y., Wang E. L., Yang X. G. & Wang J. Contributions of climatic and crop varietal changes to crop production in the North China Plain, since 1980 s. Global Change Biol 16, 2287–2299, 10.1111/j.1365-2486.2009.02077.x (2010).

[b22] Jeong S. J. *et al.* Effects of double cropping on summer climate of the North China Plain and neighbouring regions. Nature Climate Change 4, 615–619, 10.1038/Nclimate2266 (2014).

[b23] Yang N. *et al.* Effects of planting soybean in summer fallow on wheat grain yield, total N and Zn in grain and available N and Zn in soil on the Loess Plateau of China. Eur J Agron 58, 63–72, 10.1016/j.eja.2014.05.002 (2014).

[b24] Gao B. *et al.* Nitrous oxide and methane emissions from optimized and alternative cereal cropping systems on the North China Plain: A two-year field study. Sci Total Environ 472, 112–124, 10.1016/j.scitotenv.2013.11.003 (2014). 24291136

[b25] Wang X. Y. *et al.* Spatial and Temporal Variations of Crop Fertilization and Soil Fertility in the Loess Plateau in China from the 1970 s to the 2000 s. Plos One 9, ARTNe112273DOI 10.1371/journal.pone.0112273 (2014). PMC4224432 25380401

[b26] Jiang D. *et al.* Long-term effects of manure and inorganic fertilizers on yield and soil fertility for a winter wheat-maize system in Jiangsu, China. Pedosphere 16, 25–32, 10.1016/S1002-0160(06)60022-2 (2006).

[b27] Ju X. T. *et al.* Reducing environmental risk by improving N management in intensive Chinese agricultural systems. P Natl Acad Sci USA 106, 3041–3046, 10.1073/pnas.0813417106 (2009). PMC2644255 19223587

[b28] Meng Q. F. *et al.* Alternative cropping systems for sustainable water and nitrogen use in the North China Plain. Agr Ecosyst Environ 146, 93–102, 10.1016/j.agee.2011.10.015 (2012).

[b29] Liu C. M., Yu J. J. & Kendy E. Groundwater exploitation and its impact on the environment in the North China Plain. Water Int 26, 265–272 (2001).

[b30] Aeschbach-Hertig W. & Gleeson T. Regional strategies for the accelerating global problem of groundwater depletion. Nat Geosci 5, 853–861, 10.1038/Ngeo1617 (2012).

[b31] Zhang H., Wang X., You M. & Liu C. Water-yield relations and water-use efficiency of winter wheat in the North China Plain. Irrigation Science 19, 37–45 (1999).

[b32] Kang S. Z. *et al.* Effects of limited irrigation on yield and water use efficiency of winter wheat in the Loess Plateau of China. Agr Water Manage 55, 203–216 (2002).

[b33] Zhang X. Y., Chen S. Y., Sun H. Y., Wang Y. M. & Shao L. W. Water use efficiency and associated traits in winter wheat cultivars in the North China Plain. Agr Water Manage 97, 1117–1125, 10.1016/j.agwat.2009.06.003 (2010).

[b34] Qin W., Chi B. L. & Oenema O. Long-Term Monitoring of Rainfed Wheat Yield and Soil Water at the Loess Plateau Reveals Low Water Use Efficiency. Plos One 8, ARTN e78828 DOI 10.1371/journal.pone.0078828 (2013). PMC3841156 24302987

[b35] Wei Z., Paredes P., Liu Y., Chi W. W. & Pereira L. S. Modelling transpiration, soil evaporation and yield prediction of soybean in North China Plain. Agr Water Manage 147, 43–53, 10.1016/j.agwat.2014.05.004 (2015).

[b36] Paredes P. *et al.* Performance assessment of the FAO AquaCrop model for soil water, soil evaporation, biomass and yield of soybeans in North China Plain. Agr Water Manage 152, 57–71 (2015).

[b37] Paredes P., de Melo-Abreu J. P., Alves I. & Pereira L. S. Assessing the performance of the FAO AquaCrop model to estimate maize yields and water use under full and deficit irrigation with focus on model parameterization. Agr Water Manage 144, 81–97, 10.1016/j.agwat.2014.06.002 (2014).

[b38] Hua K. K., Wang D. Z., Guo X. S. & Guo Z. B. Carbon Sequestration Efficiency of Organic Amendments in a Long-Term Experiment on a Vertisol in Huang-Huai-Hai Plain, China. Plos One 9, ARTN e108594DOI 10.1371/journal.pone.0108594 (2014). PMC4180924 25265095

[b39] Lu R. K. Analytical methods of soil agricultural chemistry. China Agricultural Science and Technology Press, Beijing (2000).

[b40] Walkley A. & Black I. A. An examination of the degtjareff method for determining soil organic matter and a proposed modification of the chromic acid titration method. Soil Science 37, 29–38 (1934).

[b41] Black C. A. Methods of soil analysis. Part 2. Chemical and microbiological properties. Madison, Wisc: ASA (1965).

[b42] Dobermann, A. Nutrient use efficiency—measurement and management. In IFA, Fertilizer Best Management Practices, Brussels, Belgium. 7–9 Mar. 2007. Int. Fertilizer Industry Assoc., Paris., 1–28 (2007).

[b43] Steduto P., Hsiao T. C., Raes D. & Fereres E. AquaCrop-The FAO Crop Model to Simulate Yield Response to Water: I. Concepts and Underlying Principles. Agron J 101, 426–437, 10.2134/agronj2008.0139s (2009).

[b44] Steduto P., Hsiao T. C., Fereres E. & Raes D. FAO Irrigation and drainage paper 66, Crop yield response to water, Food and Agriculture Organization of the United Nations (2012).

[b45] Allen R. G., Pereira L. S., Raes D. & Smith M. Crop evapotranspiration. Guidelines for computing crop water requirements. FAO irrigation and drainage paper no. 56, 300 pp. (1998).

[b46] Bates D., Maechler M., Bolker B. M. & Walker S. Lme4: Linear mixed-effects models using Eigen and S4. Journal of Statistical Software (2014).

[b47] R Core Team. R: A language and environment for statistical computing *R Foundation for Statistical Computing, Vienna, Austria* URL http://www.R-project.org/(Date of access:05/03/2015) (2013).

[b48] FAO. Crop Water Information: Wheat http://www.fao.org/nr/water/cropinfo_wheat.html. (2015).

[b49] Darilek J. L. *et al.* Changes in soil fertility parameters and the environmental effects in a rapidly developing region of China. Agr Ecosyst Environ 129, 286–292, 10.1016/j.agee.2008.10.002 (2009).

[b50] Dobermann A., Cassman K. G., Mamaril C. P. & Sheehy J. E. Management of phosphorus, potassium, and sulfur in intensive, irrigated lowland rice. Field Crop Res 56, 113–138, 10.1016/S0378-4290(97)00124-X (1998).

[b51] Hoa N. M., Janssen B. H., Oenema O. & Dobermann A. Comparison of partial and complete soil K budgets under intensive rice cropping in the Mekong Delta, Vietnam. Agr Ecosyst Environ 116, 121–131, 10.1016/j.agee.2006.03.020 (2006).

[b52] Bai Z. H. *et al.* The critical soil P levels for crop yield, soil fertility and environmental safety in different soil types. Plant Soil 372, 27–37, 10.1007/s11104-013-1696-y (2013).

[b53] Daniels M. B. & Scott H. D. Water-Use Efficiency of Double-Cropped Wheat and Soybean. Agron J 83, 564–570 (1991).

[b54] Caviglia O. P., Sadras V. O. & Andrade F. H. Intensification of agriculture in the south-eastern Pampas—I. Capture and efficiency in the use of water and radiation in double-cropped wheat-soybean. Field Crop Res 87, 117–129, 10.1016/j.fcr.2003.10.002 (2004).

[b55] Singh R., Singh K. & Bhandarkar D. M. Estimation of water requirement for soybean (Glycine max) and wheat (Triticum aestivum) under vertisols of Madhya Pradesh. Indian J Agr Sci 84, 190–197 (2014).

[b56] Liu C. M., Zhang X. Y. & Zhang Y. Q. Determination of daily evaporation and evapotranspiration of winter wheat and maize by large-scale weighing lysimeter and micro-lysimeter. Agr Forest Meteorol 111, 109–120 (2002).

[b57] Kang S. Z., Gu B. J., Du T. S. & Zhang J. H. Crop coefficient and ratio of transpiration to evapotranspiration of winter wheat and maize in a semi-humid region. Agr Water Manage 59, 239–254, Pii S0378-3774(02)00150-6 (2003).

[b58] Wang P., Song X. F., Han D. M., Zhang Y. H. & Zhang B. Determination of evaporation, transpiration and deep percolation of summer corn and winter wheat after irrigation. Agr Water Manage 105, 32–37, 10.1016/j.agwat.2011.12.024 (2012).

[b59] Corbeels M., Hofman G. & Van Cleemput O. Analysis of water use by wheat grown on a cracking clay soil in a semi-arid Mediterranean environment: weather and nitrogen effects. Agr Water Manage 38, 147–167 (1998).

[b60] Lopez-Bellido R. J., Lopez-Bellido L., Benitez-Vega J. & Lopez-Bellido F. J. Tillage system, preceding crop, and nitrogen fertilizer in wheat crop: I. Soil water content. Agron J 99, 59–65, 10.2134/agronj2006.0025 (2007).

[b61] Jin L. *et al.* Evaluation of nitrogen fate, water and nitrogen use efficiencies of winter wheat in North China Plain based on model approach. Acta Agr Scand B-S P 63, 127–138, 10.1080/09064710.2014.886713 (2014).

[b62] Wang X. B. *et al.* Tillage and crop residue effects on rainfed wheat and maize production in northern China. Field Crop Res 132, 106–116, 10.1016/j.fcr.2011.09.012 (2012).

[b63] Gan Y. T. *et al.* Ridge-Furrow Mulching Systems-An Innovative Technique for Boosting Crop Productivity in Semiarid Rain-Fed Environments. Advances in Agronomy. Vol 118 118, 429–476, 10.1016/B978-0-12-405942-9.00007-4 (2013).

[b64] Evenson R. E. & Gollin D. Assessing the impact of the Green Revolution, 1960 to 2000. Science 300, 758–762, 10.1126/science.1078710 (2003). 12730592

[b65] Li S. X. *et al.* Nutrient and Water Management Effects on Crop Production, and Nutrient and Water Use Efficiency in Dryland Areas of China. Advances in Agronomy, Vol 102 102, 223–265, 10.1016/S0065-2113(09)01007-4 (2009).

[b66] Manna M. C. *et al.* Long-term effect of fertilizer and manure application on soil organic carbon storage, soil quality and yield sustainability under sub-humid and semi-arid tropical India. Field Crop Res 93, 264–280 (2005).

[b67] Cai Z. C. & Qin S. W. Dynamics of crop yields and soil organic carbon in a long-term fertilization experiment in the Huang-Huai-Hai Plain of China. Geoderma 136, 708–715, 10.1016/j.geoderma.2006.05.008 (2006).

[b68] Zhang W. J. *et al.* Relative contribution of maize and external manure amendment to soil carbon sequestration in a long-term intensive maize cropping system. Sci Rep-Uk 5 (2015). 10.1038/srep10791 PMC4454045 26039186

