# Supplementary material for: Productivity and sustainability of rainfed wheat-soybean system in the North China Plain: results from a long-term experiment and crop modelling
Source: Sci Rep. 2015 Dec 2;5:17514. doi: 10.1038/srep17514 (PMC4667244; doi:10.1038/srep17514)
Supplement: Supplementary Information [file srep17514-s1.pdf]

Productivity and sustainablity of rainfed wheat-soybean system in the North China Plain: results of a long-term experiment and crop modelling

Wei Qin [1, 4*], Daozhong Wang [1,2], Xisheng Guo[1,2*], Taiming Yang [3], Oene Oenema[4,5]

[1] *Soil and Fertilizer Research Institute, Anhui Academy of Agricultural Sciences, Hefei, 230031 China*

[2] *Key Laboratory of Nutrient Cycling and Resources Environment of Anhui Province, Hefei, China*

[3] *Anhui Center of Agricultural Meteorology, Hefei, 230031 China*

[4] *Department of Soil Quality, Wageningen UR, 6700 AA, Wageningen, the Netherlands*

[5] *Alterra, Wageningen UR, 6700 AA, Wageningen, the Netherlands*

* *Corresponding to : wei.qin@wur.nl, weiqinwur@gmail.com (W. Qin) and 1078681598@qq.com (X. Guo)*

Supplementary information

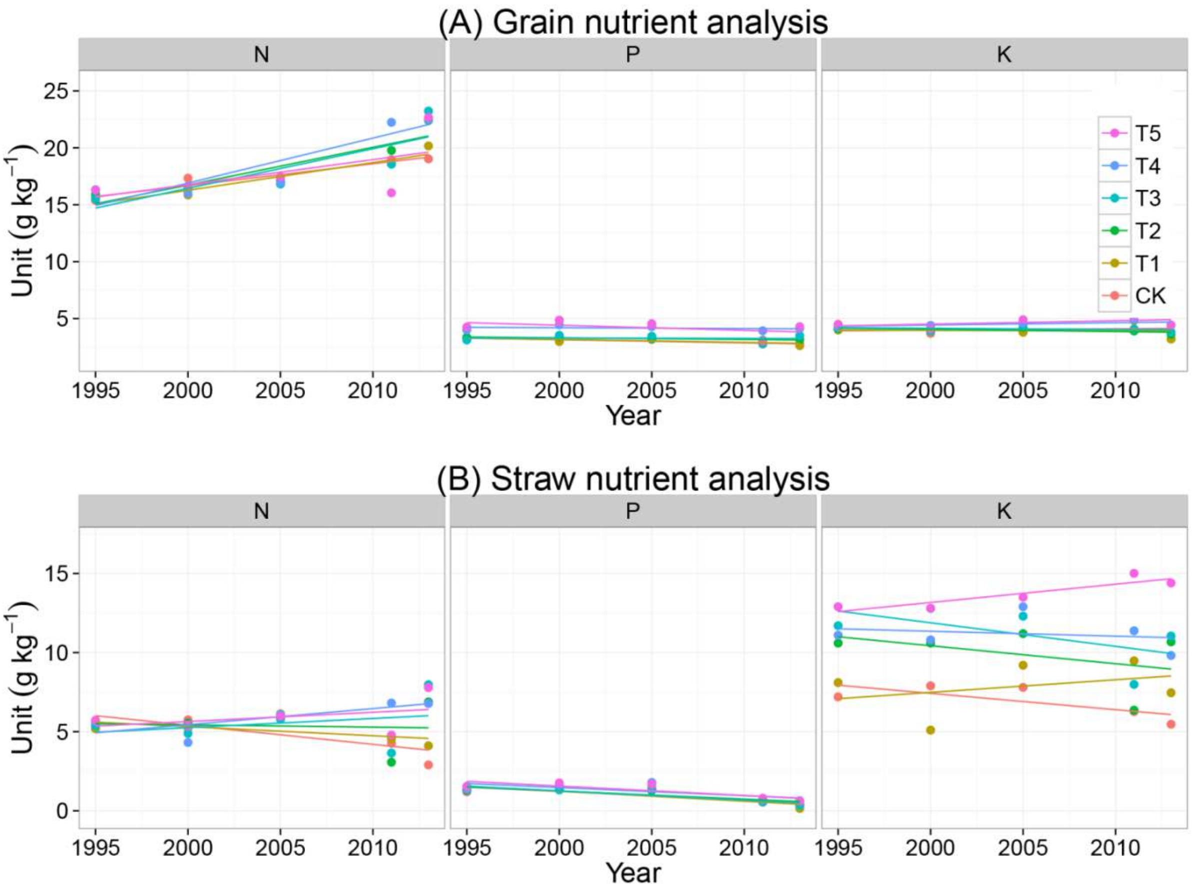

Supplementary Figure S1. Nutrient content of grain (A) and straw (B) of wheat over time. N, P and K content were analysed at harvest. Grain N content slightly increase over time, ranging from 15 to 20 g/kg, whereas grain P and K contents mostly ranged from 2.5 to 5 g/kg. Grain P and K content in manure treatments were higher than that in other treatments. Straw N content ranged from 2.5 to 7.5 g/kg, straw P content ranged from 1 to 2 g/kg and had slightly decreasing trend. Variation in straw K content was relative large (range 5 to 15 g/kg), with higher contents in manure treatments. This suggest that K was perhaps a yield limiting factor; note also that K content in CK decreased.

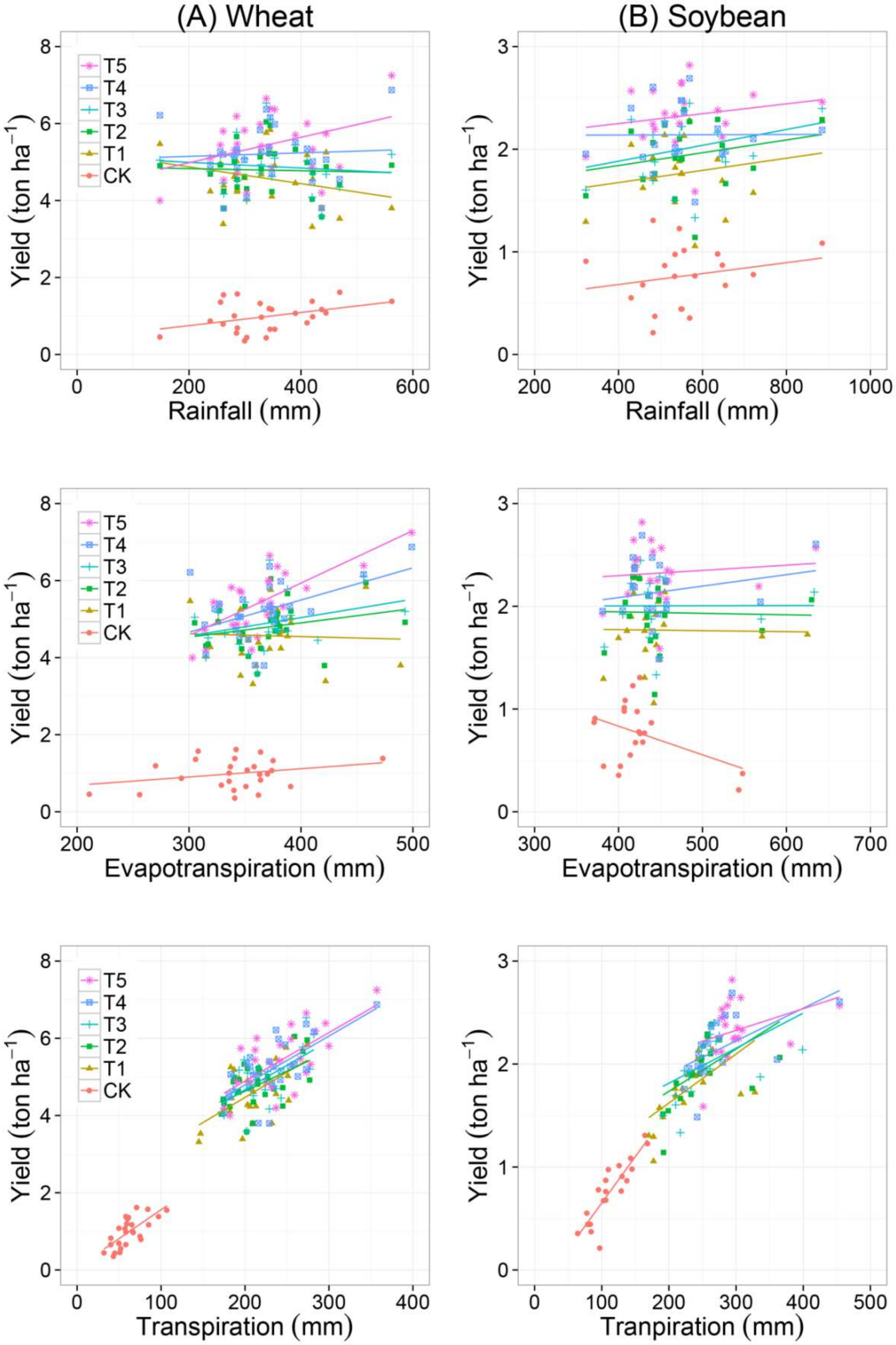

Supplementary Figure S2. Relationships between rainfall, evapotranspiration (ET) and transpiration (T) and grain yields of wheat and soybean. Statistics are summarized in Table S3.

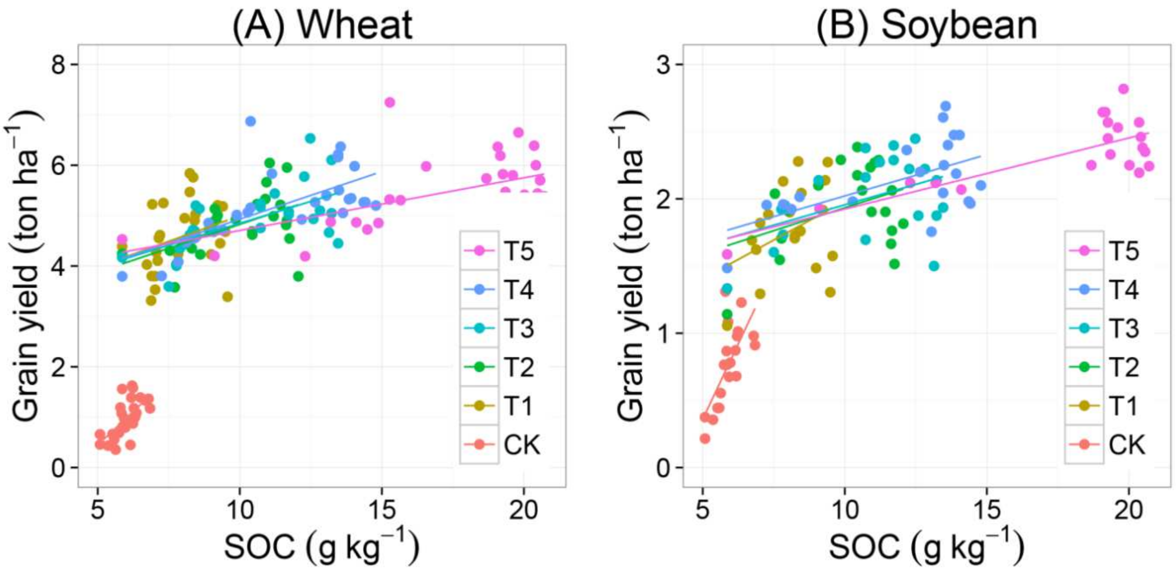

Supplementary Figure S3. Correlations between grain yields of wheat and soybean to soil organic carbon.

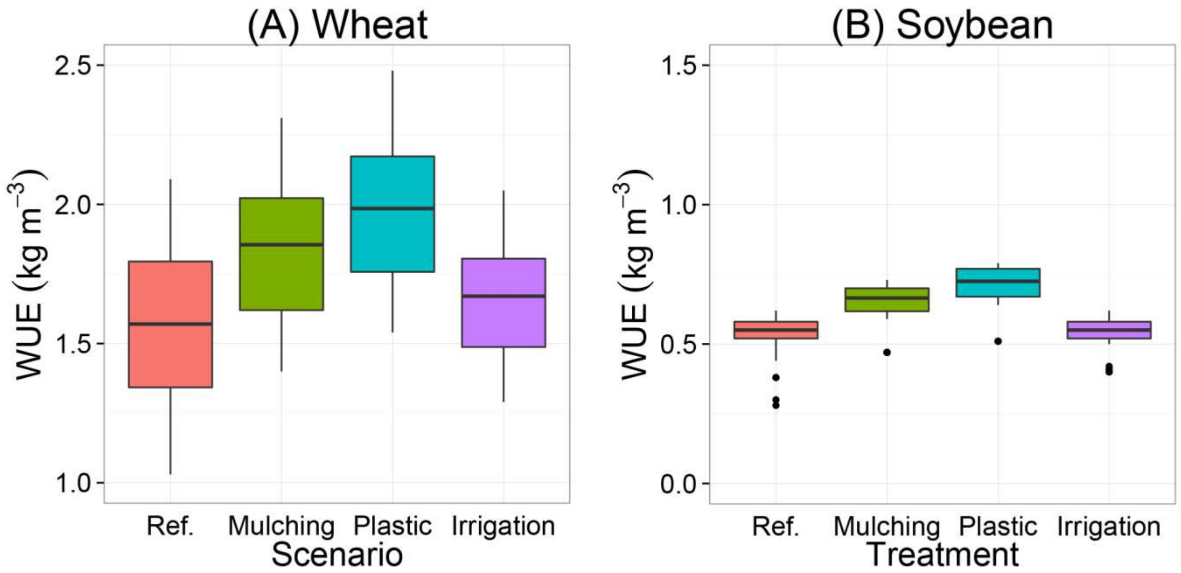

Supplementary Figure S4. Simulated WUE of wheat (A) and soybean (B) with straw mulching, plastic film cover and irrigation for the period 1982 - 2012.

Supplementary Table S1. List of crop parameters for wheat and soybean used in the AquaCrop model.

Parameters	Crops		Sources
	**Wheat**	**Soybean**	
**Crop development (in calendar days)**			
From sowing to emergence	10 days	10 days	Field observation
From sowing to max. canopy	150 days	60 days	Field observation
From sowing to flowering	160 days	75 days	Field observation
From sowing to senescence	190 days	100 days	Field observation
From sowing to maturity	220 days	110 days	Field observation
Building up Harvest Index (HI)	32 days	35 days	Field observation
Duration of flowering	15 days	10 days	Field observation
Plant density	300 plants m^{-2}	180 plants m^{-2}	Field observation
Sowing rate	150 kg seed ha^{-1}	405 kg seed ha^{-1}	Field observation
1000 seed mass	40 g	180	Field observation
Germination rate	80%	80%	Field observation
Max. root depth	2 m	2 m	Field observation
Reference HI	45%	42%	Field observation
Max HI	52%	46%	Field observation
Crop water productivity	20g m^{-2}	9 g m^{-2}	Calibrated
**Canopy development**			
Initial canopy cover	4.5%	9%	Field observation
Canopy expansion	4% day^{-1}	9.8% day^{-1}	Calculated by AquaCrop
Max. canopy cover	95%	95%	Field observation
Canopy decline	7.2% day^{-1} (41 days)	10.3% day^{-1} (28 days)	Calculated by AquaCrop
**Thresholds temperatures**			
Base temperature for biomass production	0 ^oC	0 ^oC	Calibrated
Upper temperature for biomass production	32 ^oC	35 ^oC	Calibrated
Range of cold stress for biomass production	0 - 14 ^oC	0-10 ^oC	Calibrated
Range of cold stress for pollination	4-9 ^oC	3-8 ^oC	Calibrated
Range of heat stress for pollination	32 - 37 ^oC	40-45 ^oC	Calibrated
**Water extraction pattern in the root zone**			
Upper 1/4 (0-0.5m)	40%	40%	Calibrated
Second 1/4 (0.5-1m)	30%	30%	Calibrated
Third 1/4 (1-1.5m)	20%	20%	Calibrated
bottom 1/4 (1.5-2m)	10%	10%	Calibrated
**Water stresses**			
Canopy expansion	Moderately tolerant (upper = 0.25, lower = 0.60, shape factor = 5)	Sensitive (upper = 0.15, lower = 0.65, shape factor = 3)	Calibrated
Stomatal closure	Extremely sensitive (upper=0.25, shape factor = 2.5)	Moderately sensitive (upper=0.5, shape factor = 3)	Calibrated
Early canopy senescence	Tolerant (upper = 0.75, shape factor =2.5)	Moderately tolerant (upper = 0.7, shape factor =3)	Calibrated
Aeration stress	Moderately tolerant (5 vol%)	Moderately tolerant (5 vol%)	Calibrated
**Evapotranspiration**			
Soil evaporation coefficient	Effect of canopy shelter in late season = 50%	Effect of canopy shelter in late season = 25%	Default in AquaCrop
Crop transpiration coefficient	1.1 (reduction with age = 0.15%/day)	1.1 (reduction with age = 0.3%/day)	Default in AquaCrop
**Fertilities stresses**	Considered	Considered	Calibrated

Supplementary Table S2. Results of the statistical analysis of the effects of rainfall (R), N, P and K inputs, and the interactions between rainfall and N, P and K input in wheat yields.

Interactions	Item	Estimate	Std. Error	df	t value	p value	Sign.
Included	(Intercept)	7.23	1.13	128.1	6.399	0.00	***
	β1(R)	-0.01	0.00	128.2	-3.23	0.00	**
	β2(N)	-0.01	0.02	107.7	-0.589	0.56	NS
	β3(P)	0.02	0.03	107.5	0.586	0.56	NS
	β4(K)	-8E-03	0.01	105.2	-1.438	0.15	NS
	β5(R*N)	4E-06	0.00	107.5	0.074	0.94	NS
	β6(R*P)	8E-06	0.00	107.2	0.105	0.92	NS
	β7(R*K)	4E-05	0.00	105.1	2.66	0.01	**

† Yields of the NPK treatment (T1) are set as the reference in the statistical analysis. Hence, the intercept shows the intercept of yields of T1. The following items show the effects (slopes), compared to the intercept. ‡Significance were displayed with numbers of asterisk, '***' means p value $\leq$ 0.001, '**' means 0.001< p value $\leq$ 0.01, '*' means 0.01 <p value $\leq$ 0.05 and ' NS' means p value >0.05, i.e., not significant.

Supplementary Table S3. Yield response to seasonal rainfall, evapotranspiration and transpiration.

		Rainfall					Evapotranspiration					Transpiration				
Crop	Treatment	Intercept†	Slope	r^2	p_value	Sign.	Intercept†	Slope	r^2	p_value	Sign.	Intercept†	Slope	r^2	p_value	Sign.
Wheat	CK	0.4	0.002	0.15	0.05	NS	0.3	0.002	0.07	0.18	NS	0.1	0.015	0.48	0.00	***
	T1	5.3	-0.002	0.07	0.18	NS	4.8	-0.001	0.00	0.83	NS	1.9	0.013	0.34	0.00	**
	T2	4.9	0.000	0.00	0.84	NS	3.4	0.004	0.07	0.20	NS	2.6	0.010	0.29	0.00	**
	T3	5.1	-0.001	0.01	0.62	NS	3.1	0.005	0.10	0.12	NS	2.1	0.013	0.39	0.00	***
	T4	5.1	0.000	0.00	0.80	NS	2.2	0.008	0.22	0.01	*	2.3	0.013	0.45	0.00	***
	T5	4.3	0.003	0.13	0.07	NS	0.5	0.014	0.52	0.00	***	2.4	0.013	0.43	0.00	***
Soybean	CK	0.5	0.001	0.04	0.38	NS	1.9	-0.003	0.18	0.06	NS	-0.2	0.009	0.77	0.00	***
	T1	1.4	0.001	0.05	0.37	NS	1.8	0.000	0.00	0.94	NS	0.7	0.005	0.37	0.00	**
	T2	1.6	0.001	0.05	0.33	NS	2.0	0.000	0.00	0.92	NS	0.9	0.004	0.29	0.01	*
	T3	1.6	0.001	0.08	0.22	NS	2.0	0.000	0.00	0.99	NS	1.1	0.003	0.25	0.02	*
	T4	2.1	0.000	0.00	0.98	NS	1.6	0.001	0.04	0.37	NS	1.3	0.003	0.31	0.01	*
	T5	2.1	0.000	0.04	0.40	NS	2.1	0.001	0.01	0.68	NS	1.7	0.002	0.11	0.15	NS

† The unit of intercept is ton ha^{-1}.

Supplementary Table S4. Analysis of soil total N and soil organic carbon in 2013.

					Total N (g kg^{-1})					
Treatment	0-20cm	se	20-40cm	se	40-60cm	se	60-80cm	se	80-100cm	se
CK	0.63	0.02	0.41	0.02	0.37	0.02	0.27	0.02	0.26	0.02
NPK	0.76	0.03	0.52	0.03	0.43	0.03	0.42	0.03	0.32	0.03
NPK+ wheat straw	1.10	0.02	0.54	0.04	0.52	0.05	0.42	0.05	0.34	0.05
NPK+ pig manure	1.26	0.17	0.57	0.04	0.48	0.04	0.42	0.02	0.36	0.07
NPK+ cow manure	1.72	0.18	0.71	0.05	0.65	0.09	0.48	0.11	0.41	0.04

					SOC (g kg^{-1})					
Treatment	0-20cm	se	20-40cm	se	40-60cm	se	60-80cm	se	80-100cm	se
CK	7.29	0.29	5.06	0.37	4.31	0.52	3.71	0.26	3.07	0.30
NPK	9.98	0.63	5.43	0.71	4.92	0.18	4.50	0.52	3.39	0.42
NPK+ wheat straw	12.38	0.96	6.52	0.15	6.33	0.25	4.59	0.35	3.36	0.20
NPK+ pig manure	14.64	0.47	6.63	0.44	6.00	0.58	5.70	0.85	4.39	0.23
NPK+ cow manure	21.94	0.20	7.53	0.29	7.26	0.61	5.26	0.33	4.50	0.14

Supplementary Table S5. The results of statistical analysis of Figure 4.

Items	Treatment	Intercept†	Slope	r^2	p value	Sign.‡
Soil N	CK	76.403	-0.301	0.085	0.213	NS
	T1	101.907	-0.548	0.162	0.078	NS
	T2	97.292	0.099	0.010	0.678	NS
	T3	102.285	0.175	0.031	0.460	NS
	T4	111.566	0.668	0.120	0.135	NS
	T5	115.882	1.898	0.472	0.001	***
Soil P	CK	8.064	-0.201	0.889	0.000	***
	T1	12.492	0.117	0.208	0.043	*
	T2	12.168	0.137	0.336	0.007	**
	T3	11.285	0.239	0.700	0.000	***
	T4	3.340	2.476	0.962	0.000	***
	T5	20.445	2.292	0.808	0.000	***
Soil K	CK	83.946	-0.341	0.095	0.186	NS
	T1	97.820	-0.741	0.226	0.034	*
	T2	92.679	0.668	0.080	0.227	NS
	T3	119.408	1.006	0.284	0.016	*
	T4	106.983	1.124	0.488	0.001	***
	T5	157.167	8.056	0.440	0.001	**
SOC	CK	6.620	-0.038	0.509	0.000	***
	T1	6.458	0.090	0.564	0.000	***
	T2	6.552	0.189	0.862	0.000	***
	T3	6.068	0.254	0.912	0.000	***
	T4	6.360	0.299	0.858	0.000	***
	T5	9.589	0.429	0.771	0.000	***

†The unit of intercept is ton ha^{-1}. ‡ '***' means $p < 0.001$, '**' means $0.001 \leq p \leq 0.01$, '*' means $0.01 < p < 0.05$ and ' NS' means $p \geq 0.05$, i.e., not significant.
